# Fascial Manipulation Technique in the Conservative Management of Morton’s Syndrome: A Pilot Study

**DOI:** 10.3390/ijerph18157952

**Published:** 2021-07-27

**Authors:** Carlo Biz, Carla Stecco, Ilaria Fantoni, Gianluca Aprile, Stefano Giacomini, Carmelo Pirri, Pietro Ruggieri

**Affiliations:** 1 Department of Surgery, Oncology and Gastroenterology DiSCOG, Orthopaedic Clinic, University of Padova, 35128 Padova, Italy; ilaria.fantoni89@gmail.com (I.F.); gian823@gmail.com (G.A.); pietro.ruggieri@unipd.it (P.R.); 2 Department of Neurosciences, Institute of Human Anatomy, University of Padova, 35121 Padova, Italy; carla.stecco@unipd.it (C.S.); giacste84@gmail.com (S.G.); carmelop87@hotmail.it (C.P.)

**Keywords:** Morton’s syndrome, connective tissue, deep fascia, fascial manipulation, metatarsalgia, muscle stiffness, manual therapy, non-invasive therapy

## Abstract

Background and Objective: Morton’s syndrome (MS) is a common cause of neuropathic chronic forefoot pain, characterised by the development of a swelling of the common digital plantar nerve, whose aetiology is not fully known. There is currently no gold standard of treatment; nonoperative management commonly involves manual therapies, orthoses therapy and infiltrative techniques, while surgery is indicated after failure of conservative measures. The present preliminary study prospectively evaluates patients affected by MS treated by Fascial Manipulation technique (FM), a noninvasive manual therapy, focused on the release of the deep fascia, reducing its stiffness. Materials and Methods: Patients with clinical and sonographic diagnosis of MS with at least a 4-month history of neuropathic symptoms underwent a cycle of three weekly FM sessions. Clinical follow-up, including VAS and AOFAS scores, was performed 21 days (T1) and 3 months (T2) after treatment. Results: Nine patients, among 28 recruited initially, completed the manual therapy sessions and relative follow-up points. This noninvasive pain treatment led to significant improvement of VAS (*p* = 0.0034) and AOFAS scores (*p* = 0.0240) at the first follow-up (T1). At 3-month follow-up (T2), both scores decreased slightly, remaining however superior to the pre-treatment values. Only VAS was still significant (*p* = 0.0184). Conclusions: Despite the small size of the case series, this pilot study is unique in supporting Fascial Manipulation in the nonoperative treatment of MS. Further studies are needed with a large cohort of gender balanced patients to confirm the encouraging results obtained.

## 1. Introduction

Morton’s syndrome (MS) is a common cause of neuropathic chronic forefoot pain with an estimated prevalence of 88 women in every 100,000 and 50 men in every 100,000 [1], most commonly between the fourth and sixth decade of life [2]. It is characterised by persistent painful swelling of common digital plantar nerve (CDPN), known as a “neuroma” [3,4,5]. This is actually a misnomer, as the lesion consists of a perineural fibrosis with no neoplastic tissue [6,7]. Tissue injury and forefoot pain exceeding 3 months could alter cerebral sensory processing and impair pain-inhibitory mechanisms, causing the development of chronic pain [8], resulting in a reduction of patient quality of life. The syndrome typically presents with severe burning and stabbing pain in the intermetatarsal region, which can also spread to adjacent toes, the dorsum of the foot and the hindfoot. It is worsened by walking and wearing tight-fitting shoes and is often associated with paraesthesia or dysaesthesia in the territory of the affected nerve [9,10]. Furthermore, gait modifications to reduce weightbearing on the affected foot are common [11,12]; the resulting repetitive trauma, bursitis and nerve entrapment further exacerbate symptoms [13]. Physical examination reveals pain on palpation of the affected region and a positive Mulder’s manoeuvre [14], despite lack of any specific test for determining the presence of a Morton’s neuroma. Diagnosis is confirmed by ultrasound examination or MRI, even if they demonstrate high false negative values and appear to be relevant only when the size of the neuroma exceeds 5 mm in transverse diameter [15,16]. Although its primary aetiopathogenesis is not fully known and remains controversial [17], the most common hypothesis considers MS as a canalicular syndrome [18] due to the particular conformation and functional complexity of the distal intermetatarsal region, which is a stiff osteofibrous channel. Other theories have hypothesized that the intermetatarsophalangeal bursa could play a significant role, whereas others have postulated alternative vascular or traumatic insults as a cause of the disease. Both bone structures, such as the metatarsal bones, and soft tissues, such as the dorsal metatarsal transverse ligament, the dorsal fascia and the plantar aponeurosis, can cause compression of the CDPN [19,20]. Particularly, the dorsal fascia could play a key role in nerve entrapment due to its connection with the interosseous muscles. Mechanical alterations leading to prolonged contraction of the interosseous muscles can lead to fascial dysfunction [21], resulting in increased fascial stiffness. This could cause narrowing of the intermetatarsal channel with possible entrapment of the nerve and associated evolution of a neuroma as the final manifestation [22].

In the literature, a myriad of possible therapeutic approaches for MS management is described; however, the current clinical management of MS is far from identifying a “gold standard” of treatment [2].

First-line management is nonoperative [1]. The most reliable treatments include lifestyle modifications, such as avoidance of tight fitting shoes, orthotics, local infiltrations of corticosteroids [18,23,24,25], as well as mobilisation and manipulation techniques [26,27,28,29]. Surgical intervention, recommended after failure of conservative therapies, includes neurectomy, i.e., excision of the affected CDPN segment, and neurolysis. The latter consists of a section of the dorsal fascia of the foot and the deep transverse ligament. Both techniques seem to guarantee the best outcomes for patients especially in the long term, still with a considerable probability of complications [13,14,30,31,32]. Additionally, Lee et al. [33] found that with long-term follow-up of patient outcomes (minimum of 10 years) after neuroma excision, patients demonstrate progressive worsening as compared with midterm and short-term results.

Therefore, manual therapy seems appropriate in cases of pain and potential tissue stiffness, particularly when alternative options have not provided convincingly good outcomes [27,28]. In particular, the effectiveness of the Fascial Manipulation (FM) method has already been described in other specific musculoskeletal conditions, such as non-specific low back pain [34,35,36], carpal tunnel syndrome [37] and chronic shoulder pain [38], demonstrating encouraging results in the management of those pathologies due to connective tissue dysfunctions. A recent systematic review supports the achievement of these positive clinical outcomes on patients’ pain and disability in different musculoskeletal conditions by applying this method [39]. Hence, for these reasons, the FM technique was adopted for the conservative treatment of MS. Since connective tissue and deep fascia play a key role in the aetiology of MS [20,21], and it is well-documented that nerve excision procedures risk the uncontrollable possibility of stump neuroma, pain exacerbation and permanent numbness, the rationale of the present study is that FM, acting on forefoot soft tissues, particularly on deep fascia stiffness, should be considered as a first-line non-operative treatment for this syndrome. However, as this method does not act on the neuroma itself, but only on surrounding connective tissues, no structural changes of the CDPN, such as size reduction or disappearance of the lesion, are expected.

Given these considerations and the experience in treating chronic pain due to several musculoskeletal conditions by FM [35,36,37,38,40] this pilot study is aimed at evaluating the effectiveness of the FM technique focused on the release of the deep fascia as a nonoperative therapy for MS in terms of pain reduction and functional recovery.

Our study hypothesis was that the fascial release provided by FM could reduce the stiffness of the intermetatarsal space and decrease CDPN compression with consequent improvement of the neurological symptoms. Finally, to the best of our knowledge, our experience is unique because no previous clinical trials have used FM for the conservative treatment of MS.

## 2. Materials and Methods

### 2.1. Patient Selection

In this single-centre pilot study, patients with a mean age of 62 ± 21 years (range, 40–82 years), clinical history and confirmation of MS by dynamic ultrasound diagnosis were prospectively and consecutively enrolled before evaluating their outcomes.

Their demographics, including age, sex and clinical data were recorded from January 2018 to June 2019 at our level-1 healthcare trauma centre, a multi-disciplinary and multi-specialty regional university hospital. All patients participating in the study received a thorough explanation of the risks and benefits of inclusion and gave their oral and written informed consent to publish the data. This study was approved by the Institutional Ethics Committee (protocol number 3355/AO/14, approved on 29 January 2015).

**Figure 1:**
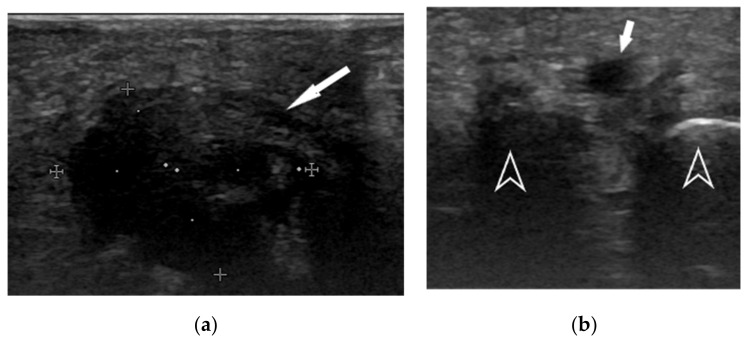
Dynamic ultrasound (**a**) showing a neuroma of the III intermetatarsal space (arrow). During Mulder’s test (**b**), the lesion (arrow) is pushed superficially, thus becoming apparent between the adjacent metatarsal heads (arrow heads).

For this analysis, patients with diagnosis of MS after dynamic ultrasound assessment had to match the following inclusion criteria: at least a 4-month history of chronic pain and neuropathic symptoms, pain on palpation of either the second or third intermetatarsal space, a positive Mulder’s click test and a positive digital nerve stretch test. These tests and dynamic ultrasound diagnosis (Figure 1 and Video S1) were considered primary inclusion criteria for this study.

Exclusion criteria were as follows: concomitant presence of the most common forefoot deformities (severe hallux valgus or rigidus; fixed and inextensible lesser toe deformities; bunionette of the fifth metatarsal bone); previous infiltrative treatment or alcoholisation, previous surgical interventions to the involved foot, multiple neuromas, diabetes mellitus, neuropathies, uncooperative patients and psychiatric diseases.

### 2.2. Fascial Manipulation

Fascial Manipulation is a manual therapy focused on the deep muscular fascia, involving deep digital pressure exerted over specific points defined by the biomechanical model of the method. This biomechanical model, developed by Luigi Stecco, describes the fascia not just as an idle membrane but more like a three-dimensional continuum, characterised by definite relationships with the underlying muscles. This dynamic structure assumes a coordinating role for motor units, merged in the more complete “myofascial unit”. Many adjacent and unidirectional myofascial units develop a “myofascial sequence” [41,42]. Hence, the selection of the points is according to precise clinical examination as indicated by Fascial Manipulation guidelines [41]. The choice of the number and specific sites for manipulation is made by the operator on the basis of a tailored analysis of each individual patient’s needs.

Fascial dysfunctions have been implied in various clinical conditions, such as abnormal proprioception, alterations of mechanical coordination, balance and pain [43,44,45].

For these reasons, it is important to recognize that different modalities of approach have to be taken into consideration for planning treatment.

**Figure 2:**
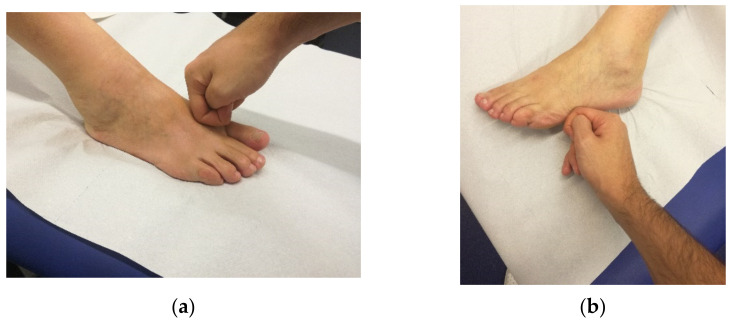
Example of manual friction using knuckles applied on ANTE-PES (**a**) and RETRO-PES (**b**) points during the FM session of a patient affected by MS.

The analytical approach of FM produces a personalised treatment for each patient: a combination of specific movements and palpatory tests allows the therapist to identify the sequences involved in the connective tissue dysfunction. The treatment is applied to a specific area of the involved myofascial sequence, known as the centre of coordination (CC) [46], where the gliding of the subcutis should be restored if altered. A localised deep friction over CCs causes hyperaemia that can modify the extracellular matrix and restore fascial gliding, leading to immediate pain reduction and increased range of motion of the suffering joint, which can be objectively evaluated by the therapist [47,48]. CCs are often at a distance from the actual site of pain, allowing FM to be applied safely even during the acute phase of an impairment. Treatment is modulated in relation to the stiffness/lack of gliding perceived over the CCs and the pain acknowledged by the patient. According to FM guidelines [46], CCs need to be manipulated until the perceived alteration in gliding has almost disappeared (in about 3 min), the patient’s pain has decreased by 60% with respect to the beginning of the treatment and any referred pain has disappeared (Figure 2).

### 2.3. Treatment Protocol

The adopted protocol involved a survey phase first, followed by a three-step treatment, which the patients underwent from January to July 2019 at our institution. The survey consisted of a short interview regarding recent and past medical history to identify any possible pathogenic factors. Particular attention was paid to previous injuries with a problematical recovery, overuse conditions, chronic and neuropathic pain and internal dysfunctions.

Clinical examination included palpation of the involved webspace and Mulder’s test; these parameters, other than for diagnostic confirmation, were obtained to evaluate the inflammatory state of the area. All preliminary data were obtained by the authors (I.F. and G.A.), not directly involved in the treatment.

After this phase, three weekly sessions of FM treatment were provided. All treatments were performed by a registered practitioner physiotherapist, certified for the Stecco method (S.G.) with more than 10 years of experience in the technique of FM, unaware of the study protocol, not involved in the investigations and compilation of questionnaires nor in data analysis. Especially in the first phase, the treatment was done far away from the site of pain, following the anatomical continuity of the fascial structures and the current characteristics of the patients, as the treatment of an oedematous and painful foot would have been useless and extremely uncomfortable for the patient. Consequently, in cases of marked local inflammation, the first treatment involved myofascial sequences proximal to the inflamed area. After a week, before the second physiotherapy session, the operator made a quick re-evaluation of both the foot and treated myofascial points to adapt the treatment according to patient feedback. The third session followed the same practice.

To test the effectiveness of the FM method on symptomatic patients, no form of foot-care education was provided to them during the treatment period and their follow-up; neither were specific changes in their daily life or physical activity level suggested. However, because of the persistent pain on their forefoot, they were used to wearing comfortable shoes, generally sporty.

### 2.4. Patient Evaluation

From January to October 2019, the clinical analyses were carried out by two independent investigators who were not directly involved in the patients’ treatment. The visual analogue pain scale (VAS) and the American Orthopaedic Foot and Ankle Society (AOFAS) questionnaire [49] were administered prior to the first treatment (T0) and post-treatment: both 21 days (T1) and 3 months (T2) after the last session. VAS consists of a straight line (10 cm) with the endpoints defining extreme limits such as ‘no pain at all’ (0 cm) and ‘worst pain’ (10 cm) to measure pain intensity [50]. AOFAS score is composed of nine questions for a total of 100 points and covers three categories: pain (40 points), function (50 points) and forefoot alignment (10 points). A higher score indicates better quality of life. No surveys were performed during intermediate phases since the condition of temporary iatrogenic inflammation associated with the treatment could have altered the results.

### 2.5. Statistical Analysis

Statistical analysis was performed by an independent statistician. The mean value and standard deviation (SD) of the measurements were considered the representative estimators of the scores. Normal distributions were tested using the Kolmogorov–Smirnov test. Differences in the VAS scale and in the AOFAS questionnaire at different time points were statistically analysed by the Kruskal–Wallis test followed by Dunn’s multiple comparisons test. All of the analyses were performed using GraphPad PRISM 3.03 (GraphPad Software Inc., San Diego, CA, USA), and *p* < 0.05 was considered the threshold for statistical significance.

## 3. Results

During a 15-month period, 28 Caucasian patients (28 feet) with diagnosis of chronic pain due to MS confirmed by dynamic ultrasound met the inclusion and exclusion criteria of this study. However, we could not evaluate 19 patients as 11 refused to participate in FM sessions, while 8 completed only one of the three steps scheduled in the study treatment protocol.

Hence, 9 patients, 1 male and 8 females, with a mean age of 62 ± 21 years (range, 40–82 years) were enrolled in the study. An average symptom duration of 23 months (range 4–60) was recorded. The neuroma was located in the left foot in seven patients and in the right foot in two cases. The second intermetatarsal space was involved in one case, while in the remaining cases the third space was affected. No cases of bilateral neuroma were recorded. In 6 patients, the diameter of the lesion exceeded 5 mm upon ultrasound examination with an average dimension of 5.9 ± 2 mm.

The foot was the region with the greatest number of treated points (53 out of 143). However, the majority of points were located outside the foot, both in the lower limb (27 points in the leg, 28 in the knee region, 10 in the thigh), in the pelvis (16 points) and in the lumbar region (9 points). The distribution of the treated points is reported in Table 1.

**Figure 3:**
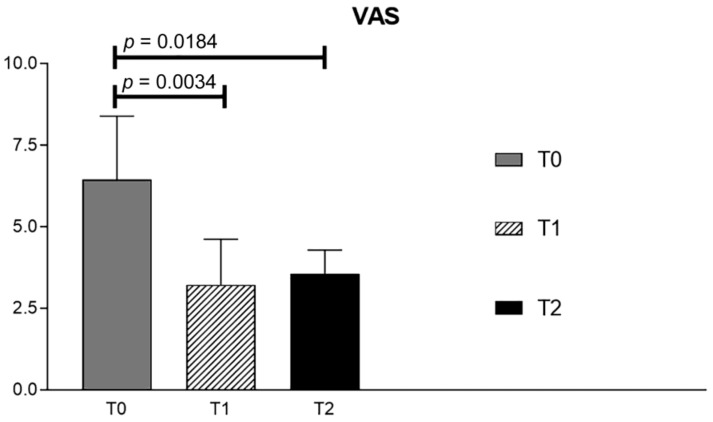
VAS values before treatment (T0), after treatment at 21 days (T1) and at 3-months follow-up (T2).

An average pre-treatment VAS (T0) of 6.429 was observed in the treated patients. After treatment (T1), the VAS decreased to a mean value of 3.143, showing a statistically significant difference with the previous (*p* = 0.0034). Three months after treatment (T2), the average value was 3.714, remaining significantly lower than the pre-treatment value (*p* = 0.0184). No significant difference was found between the T0 values and the results at T3. Data are shown in Figure 3 and Table 2.

**Figure 4:**
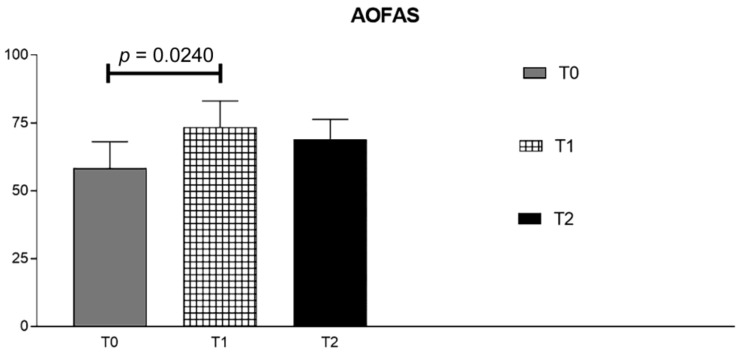
AOFAS values before treatment (T0), 21 days after treatment (T1) and at 3-months follow-up (T2).

Before treatment (T0), an average AOFAS score of 58.29 was found. At the first evaluation after treatment (T1), the score reached a mean value of 73.43, with significant improvement (*p* = 0.0240). After 3 months (T3), the average score decreased to 69 points, remaining higher than pre-treatment (*p* = 0.2957), although showing no significant difference compared to pre-treatment. Results are shown in Figure 4 and Table 3.

## 4. Discussion

Intermetatarsal neuropathy is known as a common cause of forefoot chronic pain for which several therapies, both invasive and noninvasive, have been proposed in the literature [2,11,25,27,28]. Among the latter, physical therapy aims at improving strength, flexibility and balance in patients suffering from neuropathic chronic pain, although it is usually under-utilized by therapists in general common practice, probably because of a lack of supporting evidence [28,51].

Despite the already described effectiveness of the FM method in other specific musculoskeletal conditions and connective tissue dysfunctions especially in preventing chronicity [35,52], no previous application of this method has been described for the conservative management of MS. Hence, the purpose of this prospective, pilot study was to describe and evaluate the effective application of the FM technique in the conservative management of patients with chronic pain affected by MS, after confirmation of neuroma by forefoot dynamic ultrasound.

Although further research will be required to prove the initial hypothesis of the study definitively, our preliminary findings suggest that FM was effective for chronic pain control in the short term (T1) with significant VAS reduction after treatment (*p* = 0.0034) and for the improvement of foot function, as shown by the significant increase of the AOFAS score (*p* = 0.0240). As the fascial tissue is the exclusive target of the applied treatment, it can be considered the only variable affected by this technique. The efficacy of the treatment further supports the previous hypothesis of a fascial involvement in CDPN compression and in MS development, thus giving a rationale to the use of this manual therapy aimed at relieving fascial tension and associated neuropathic symptoms [22].

Even if the clinical evaluation after 3 months (T2) showed that the results tend to decrease with time, particularly regarding foot function, the FM technique could be considered an important aid to treat neuropathic pain, especially as part of a more comprehensive plan, representing a valid alternative to the heterogenous group of conservative methods (local injection with corticosteroids and local anaesthetics, orthotics, general physiotherapy) already described in the literature [1,11,23,25]. What is most important to note, is that there were no cases of symptoms worsening with respect to the pre-treatment values, and no adverse reactions due to the treatment protocol were recorded.

Hence, in agreement with other authors [53], we believe there is the need for all symptomatic patients to have a trial of nonoperative management before proceeding with operative intervention. In particular, a target of this treatment could be patients not suitable for surgery due to medical comorbidities, those patients for whom local injections with corticosteroids and anaesthetics are contraindicated (e.g., diabetics) or simply those who prefer to avoid surgical treatment for a symptomatic neuroma with a transverse diameter less than 5 mm. Finally, in patients waiting for surgery, this technique could be employed for pain management before operative excision of neuroma.

**Figure 5:**
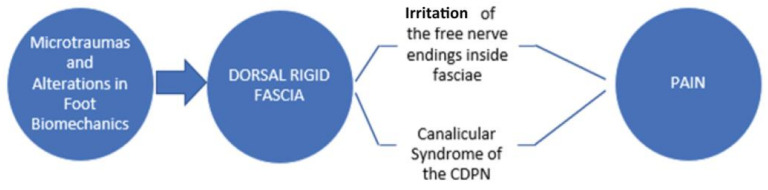
Hypothesis of a role of deep fascia in the pathogenesis of MS.

These effects of FM of pain treatment are strengthened also by the fact that the deep fasciae are very well innervated [54], and consequently, their alteration can be a further source of neuropathic chronic pain. Stiffness of fascia in the foot, other than leading to CDPN entrapment and MS development, could also irritate the free nerve endings inside the fascial tissue, causing an additional source of nociceptive pain (Figure 5) [21,55].

Another favourable aspect of FM is related to the localisation of the treated points. A common issue of other manual therapies already discussed in the literature is that treatment is mostly focused on the foot. Sault et al., for instance, described the case of a patient with MS treated by multiple grade IV mobilisations of hindfoot and midfoot joints, with improvement of neuropathic symptoms [28]. Cashley et al. reported significant pain decrease after the use of a single thrust procedure of the affected metatarsophalangeal joint [27]. Perez-Dominguez and Casaňa-Granell combined soft-tissue massage in the lower leg and foot region with grade IV mobilisations and active exercises for joint mobilisation and muscle strengthening, showing pain decrease and functional improvement [29]. All of these techniques focus on a region that can be highly painful and intractable in the acute phase, making treatment more complex, especially in the initial setting. On the contrary, many points were located in the inferior limbs in this study, not just in the foot. In the majority of cases, the treatment of the foot fasciae was avoided in the first session because it would have been too painful; instead, pain relief was often obtained without touching the most involved area. This is possible because of the fascial organisation that creates continuity throughout the body. The deep fascia of the inferior limbs can be compared to a sock, where the foot is the final portion of a more global vision. By releasing fascial tension in the leg or thigh, the technique is able to decrease fascial stiffness also in the foot because of these anatomical continuities. Due to this specific approach, FM can also be used during the acute inflammatory phase of the connective tissue.

It is important to note that other authors also considered a treatment strategy based on the release of the CDPN. Recently, Elghazy et al. [18] proposed a surgical technique for the open release of intermetatarsal ligaments (IML), resulting in nerve decompression by alleviating both IML incarceration and metatarsal head compression via expansion of the available intermetatarsal space in both the transverse and sagittal planes. In their retrospective, 12-case series studies, these authors reported equal or better clinical outcomes at short-term follow-up than those reported for nerve excision, considering that the complications reported after nerve removal do not appear to exist with simple IML decompression. Finally, they argued that if symptoms persist in the patients after IML release, the option for neuroma excision still remains. In contrast, when a nerve has already been removed and symptoms remain, no further surgical intervention is possible. Similarly, as supported by our preliminary results, FM could be proposed as a valid alternative to CDPN excision as a potential definitive treatment continuing with the physiotherapy sessions or in the pending period for the operation to mitigate the neurological symptoms.

The diagnosis of MS is usually clinical, based on the patient’s history and forefoot examination. In this study however, diagnosis was confirmed by dynamic ultrasound because it is simple, unexpensive and reliable in the presence of an experienced radiologist despite its high dependence on the operator [56]. Moreover, it is a fast and well-tolerated technique with virtually no contraindications, providing a clear correlation between neurological symptoms and localisation of the nerve lesion [23,57]. For these reasons, ultrasound can be considered superior to MRI for diagnosis of MS [16]. To the best of our knowledge, this is the first prospective, single-centre, preliminary case series study evaluating the role of the FM technique performed by a single experienced physical therapist for the nonoperative management of patients with chronic pain affected by MS in terms of pain reduction and functional improvement.

### Limitations

This pilot study has several limitations. The number of evaluated patients was limited to 9 at final follow-up because of the patients who chose not to participate, and this may have influenced the outcomes. This is partly due to the difficulty of recruiting chronic pain patients with isolated clinical and ultrasound diagnosis, including positive Mulder’s click test, of MS not being associated with the most common forefoot deformities (severe hallux valgus or rigidus, fixed and inextensible lesser toe deformities, bunionette of the fifth metatarsal bone) despite the diffusion of this canalicular syndrome. Further, some of our initially selected patients refused to undergo FM treatment because of misleading beliefs about the possible pain of manual therapy. Symptomatic patients without sonographic examination were excluded to minimise the risk of enrolling false positive patients. Nevertheless, the number of patients in our study is similar to or higher than that in some recent reports for manual therapy of Morton’s neuroma.

Further, two other aspects of our study protocol could have affected the potential number of participants recruited: constant presence of the chronic pain of at least 4 months instead of 3, as generally defined, and a positive Mulder’s click test. However, the authors believe the sample would not have increased appreciably. First, a cut-off of 4 months of chronic pain was established because patients generally turn to the orthopaedic consultant after several months of pain, initially being not well localised in the forefoot and perceived more as burning or discomfort rather than a true pain. Often, the symptoms are not continuous, as the patient benefits from the summer period in wearing wide shoes; while the pain becomes stronger again during autumn when patients start wearing stiffer footwear. Second, the presence of the Mulder test in our clinical practice is fundamental in the clinical differential diagnosis to distinguish neuroma, bursitis or metatarsalgia. Thus, the symptoms and clinical signs had to be very specific for Morton neuroma to allow the potential participants to be included in this pilot study.

Moreover, patients treated by orthoses with forefoot modification before the enrolment were not excluded by the original study protocol. However, none of the 9 out of 28 patients evaluated used orthoses previously.

Although no foot-care education was provided to the participants during the treatment period and their follow-up, to test the effectiveness of the treatment, they were used to wearing comfortable footwear. However, this aspect may have only marginally influenced our results, as patients used to wear these even before their enrolment.

Another limitation of the present report is the lack of specific scores dedicated to myofascial pain and of a clear diagnostic test to confirm and quantify the involvement of fasciae in MS, which partially impair results assessment. AOFAS is a technical score mainly employed for evaluating clinical outcomes after foot surgery, which also considers structural alterations of the forefoot, such as severe toe deformities, forefoot alignment and joint stiffness or instability [58]. As these aspects cannot be changed by FM, they were considered as patient exclusion criteria. To avoid room for treatment expectation bias, AOFAS and VAS scores were all taken by an independent researcher other than the treating clinician. Certainly, the development of scales specific for fascial disorders is needed to better characterise the effects of this manual therapy on MS.

## 5. Conclusions

Despite the small number of patients recruited, this preliminary study supports the effective application of Fascial Manipulation in the nonoperative treatment of MS, showing significant reduction of chronic pain and improvement of neuropathic symptoms at least in the short term. As no high-quality evidence currently exists to indicate which intervention should be the gold standard for non-invasive treatments, this innovative physical therapy protocol could be considered an alternative to conservative methods already proposed before proceeding with operative intervention. However, further studies are needed to evaluate the medium- and long-term effects of the protocol, which will have to be extended to more FM sessions, and to improve the generalisability of our encouraging findings on a larger group of patients affected by MS. Its future major advantages could be not only potentially resolving pain with equal efficacy as CDPN excision, but also doing so without incurring the additional potential risks of stump neuroma formation or permanent numbness.

## Figures and Tables

**Table 1:** Distribution of treated points.

Treated District	Number of Treated Points
Back	9
Pelvis	16
Thigh	10
Knee	28
Leg	27
Foot	53

**Table 2:** VAS values before treatment (T0), after treatment at 21 days (T1) and at 3-months follow-up (T2).

	T0-VAS	T1-VAS	T2-VAS
**Mean**	6.444	3.222	3.556
Std. Deviation	1.944	1.394	0.7265
Std. Error of Mean	0.6479	0.4648	0.2422
Lower 95% CI of mean	4.95	2.15	2.997
Upper 95% CI of mean	7.938	4.294	4.114
Coefficient of variation	30.16%	43.28%	20.43%

**Table 3:** AOFAS results before treatment (T0), 21 days after treatment (T1) and at 3-months follow-up (T2).

	T0-OFAS	T1-AOFAS	T2-AOFAS
**Mean**	58.29	73.43	69
Std. Deviation	9.827	9.641	7.326
Std. Error of Mean	3.714	3.644	2.769
Lower 95% CI of mean	49.2	64.51	62.22
Upper 95% CI of mean	67.37	82.35	75.78
Coefficient of variation	16.86%	13.13%	10.62%

## Data Availability

The data presented in this study are available on request from the corresponding author.

## Supplementary Materials

The following are available online at https://www.mdpi.com/article/10.3390/ijerph18157952/s1, Video S1: Dynamic ultrasound showing Morton neuroma in the third intermetatarsal space in a female patient with a positive Mulder’s click test and a positive digital nerve stretch test.
